# Colorimetric and Fluorometric Determination of Fluoride in Tetrahydrofuran and Dimethyl Sulfoxide Using a 4-Hydroxypyrene Probe

**DOI:** 10.1155/2024/5566082

**Published:** 2024-07-26

**Authors:** Yue Sun, Yunchen Long, Wenhao Sun, Yibo Zhang, Qianhui Tang, Chan Li, Sihua Li, Jing Nie

**Affiliations:** ^1^ Institute of Building Intelligence Jiangsu Vocational Institute of Architectural Technology, Xuzhou 221116, Jiangsu, China; ^2^ School of Chemical Engineering and Technology China University of Mining and Technology, Xuzhou 221116, Jiangsu, China; ^3^ Department of Materials Science and Engineering City University of Hong Kong, Hong Kong, China; ^4^ Environmental Engineering Dalian Ocean University, Dalian 116023, Liaoning, China

## Abstract

F^–^ ions (fluoride ions) are crucial in various chemical waste and environmental safety contexts. However, excessive fluoride exposure can pose a threat to human well-being. In this study, a simple 4-substituted pyrene derivative known as 4-hydroxypyrene (**4-PyOH**) was designed as a colorimetric probe for detecting F^–^ through the formation of hydrogen bonds between F^–^ and a hydroxyl group. The probe **4-PyOH** exhibited exceptional sensitivity and selectivity towards F^–^ ions and was successfully utilized as test strips for detecting F^–^ ions in organic solvents. The detection limit reached an impressively low level of 3.06 × 10^−7^ M in the organic solvent. The recognition mechanism was confirmed through ^1^H NMR titration.

## 1. Introduction

Pyrene is recognized as one of the paramount fluorophores owing to its remarkable chemical and photophysical characteristics. Notably, it exhibits substantial Stokes shifts and high quantum yields, which have garnered enduring interest in scientific circles [1–4]. In addition, pyrene can be readily modified by specific chemical modifications to improve their photophysical properties [5–7]. Pyrene-based fluorescent probes have widely been used to detect various ions, small molecules, and reactive species [8–13]. However, research on pyrene has primarily concentrated on the first position, while investigations regarding the second and fourth positions are relatively limited [14, 15]. The properties of pyrene are influenced not only by various substitution groups but also by the substituents at the generation position. This indicates that unorthodox substitution of pyrene holds significant research value.

Fluorescence sensors are often the preferred choice for measuring F^−^ compared with the traditional detection techniques attributed to their low cost, simple operation, high sensitivity, and rapid response [16–18]. Recent literature suggests that the F^−^ probes mainly include the following four mechanisms [19–22]: hydrogen bonding between F^−^ and hydroxy or amino group in the sensor, the chemical reaction between boron and F^−^, F^−^ Lewis acid interactions, and F^−^ induced cleavage reactions. Among the mechanisms above, many reported F^−^ probes are based on the hydrogen bonding mechanism, where F^−^ induced deprotonation of hydroxy groups leads to the formation of phenolate anions and distinct color and fluorescence changes. Up to now, there have been many reports of F^−^ detection based on naphthalimide, rhodamine, borondipyrromethene (BODIPY), etc. Nevertheless, few researchers pay attention to applying pyrene-based fluorescent probes for F^−^ (see Table S1 in support information). Hence, our research aims to develop a highly sensitive pyrene-based fluorescent probe to detect F^−^.

Fluorine and fluoride are widely used in various industrial fields [23, 24], and fluorine additives are significant solutes in the same electrolyte [25]. However, when lithium batteries are dismantled, the resulting electrolyte waste can be released into the environment, leading to compounds containing fluorine, arsenic, and phosphorus [26, 27]. Moreover, organic solvents, such as tetrahydrofuran and dimethylsulfoxide, are of paramount importance as the primary liquid components in lithium batteries, contributing significantly to the overall performance and efficiency of the batteries [28, 29]. The electronic semiconductor industry also utilizes hydrofluoric acid during etching [30–32]. Similarly, the chemical fertilizer, pesticide, chemical, and petrochemical industries either employ fluorine-containing chemicals or generate fluorine-containing chemical by-products [33]. Consequently, fluorine contamination caused by the industrial related wastes poses a threat to the environment as well as the individual's health and safety of working factory. Given the above thinking, monitoring and easy-to-read detection of fluoride ions have become a necessary and very important research study.

This study successfully designed and synthesized a novel fluorescence probe called **4-PyOH** based on the pyrene structure. The probe utilized the phenolic –OH group as a recognition site for the rapid and highly sensitive detection of F⁻. The design strategy focused on introducing a hydroxyl group at the 4-position of the pyrene molecule, as the electron cloud density at this position was lower than the 1-position. Consequently, the hydroxyl group in **4-PyOH** exhibits increased acidity, making it more prone to deprotonation and generating its phenolate anion. The phenolate anion possesses distinct photophysical properties, which enable effective and efficient fluorescence detection of fluoride ions.

## 2. Experimental

### 2.1. Materials and Instrumentation

The tetrahydrofuran (THF) and dimethylsulfoxide (DMSO) used in the spectral analysis were purchased at the high-performance liquid chromatography (HPLC) level and dried to remove the dissolved water before use.

The chemicals (pyrene, DDQ (4,5-dichloro-3,6-dioxocyclohexa-1,4-diene-1,2-dicarbonitrile), Na, Br_2_, CuI, HBr (33 wt.% in acetic acid), CH_3_ONa, and acetic acid (HAc, 99.5%)) used in this work were purchased commercially (energy chemical, China). Unless emphasized, all other solvents and reagents were commercially available and used without further purification. Deionized water was used for preparing samples, buffer solutions, and other experimental procedures. Mass spectra were obtained on Bruker ultrafleXtreme MALDI TOF (matrix-assisted laser desorption ionization–time-of-flight mass spectrometry) mass spectrometer. The fluorescence emission spectra were measured by a Hitachi fluorescence spectrometer (F-4600) (Ex: 365 nm). UV-VIS-NIR (ultraviolet-visible-near-infrared) spectrophotometer was used to obtain UV-vis spectra on Shimadzu UV-3600. ^1^H-NMR (^1^H-nuclear magnetic resonance) spectra and ^13^C-NMR (^13^C-nuclear magnetic resonance) spectra of compounds were obtained from Bruker-600 MHz instrument with TMS (tetramethylsilane) as the internal standard.

### 2.2. Synthesis

**Scheme 1:**
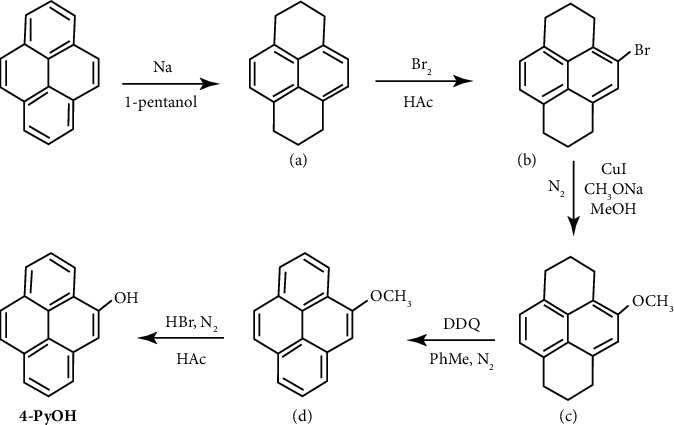
The synthesis of compound **4-PyOH**.

The detailed steps for synthesizing probe **4-PyOH** in Scheme 1 have been reported in a previously published paper [14]. Briefly, commercial pyrene was hydrogenated to form 1,2,3,6,7,8-hexahydropyrene (compound **a**), which was then converted into a 4-bromo-substituted pyrene-based compound **b** in solvent HAc. Subsequently, intermediate **b** underwent methoxylation catalyzed by CuI, resulting in the formation of compound **c**. In the following step, compound c was easily transformed into compound **d** through dehydrogenation oxidation in the presence of DDQ, yielding a high yield of 90%. Finally, the desired **4-PyOH** was synthesized by classical demethylation of the methoxyl group with a yield of nearly 100%.

### 2.3. General Procedure for Optical Studies

Probe **4-PyOH** was dissolved individually in dry THF and DMSO to prepare stock solutions with a concentration of 1 mM. The stock solutions of F⁻ were prepared separately using tetra-n-butylammonium fluoride (TBAF) in dry THF and DMSO at a concentration of 1 mM. Subsequently, the stock solutions were appropriately diluted to a suitable concentration for the experimental procedure. The fluorescence measurements were conducted at room temperature using an excitation wavelength of 365 nm, with both the excitation and emission slits set at 5 nm.

### 2.4. ^1^H-NMR Experiments

Four nuclear magnetic tubes were each loaded with 6 mg of **4-PyOH** and 0.6 mL of *d*_6_-DMSO to facilitate dissolution. TBAF solutions at 0 eq, 1.0 eq, 2.0 eq, and 3.0 eq equivalents were sequentially added to the respective solutions. After 10-minute incubation at room temperature, 1H-NMR spectra were acquired for analysis.

### 2.5. Paper Strips

To facilitate the portable application of probe **4-PyOH**, the test papers were prepared by immersing filter papers with unified shapes in dichloromethane (DCM) solution of **4-PyOH** (2 mM) for 5 minutes and then drying those test papers under a nitrogen atmosphere at room temperature for 10 h. For the detection of F^−^, the prepared test papers were dipped into dried THF solution with different concentrations of TBAF (0 mmol/L, 1 mmol/L, 5 mmol/L, 10 mmol/L, 50 mmol/L, 100 mmol/L, and 200 mmol/L).

## 3. Results and Discussion

### 3.1. Fluorine Ion Sensing Properties of Probe **4-PyOH**

**Figure 1:**
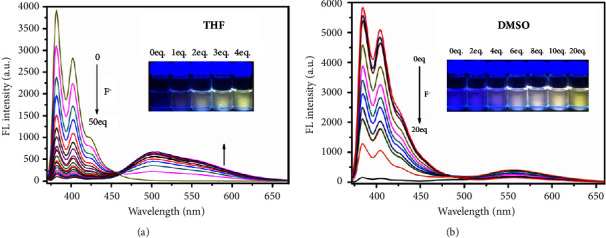
Fluorescence spectra of **4-PyOH** (10 *μ*M) in THF (a) and DMSO (b) in the presence of different concentrations of F^−^ (0−500 *μ*M and 0−200 *μ*M, respectively). Insert images: fluorescence photographs of **4-PyOH** (10 *μ*M) in THF and DMSO with different concentrations of F^−^ under illumination with a 365 nm UV handhold lamp.

The fluorescence spectra of **4-PyOH** were measured with the increase of F^−^ concentration by titration experiments. As shown in Figure 1, with the gradual increase of F^–^ content, the emission peak at 382 nm gradually decreased, while the emission peak at 502 nm gradually increased (in the dry THF). At the same time, the fluorescent emission gradually shifted from blue to yellow. Similar phenomena were observed when detecting the F^–^ in dry DMSO; however, its sensitivity was not as fast as in THF and color changes were slower than in THF as well. These phenomena indicated that the probe could be applied to detecting F^–^ in organic waste with high sensitivity and readable convenience. To further study the sensor properties of the probe for F^–^, titration experiments were carried out in dried DMSO and the THF (Figure 1). After three parallel tests, the detection limits were calculated based on the fitting results (Figure S1) and the formula [34]: detection limit = 3*σ*/*k*.

*σ* represents the standard deviation of the blank sample, while **k** denotes the slope of the relationship between the fluorescence intensity ratio and the concentration of F^−^. According to the formula, the detection limits of the probe were calculated to be 3.06 × 10^−7^ M in THF solution and 9.82 × 10^−7^ M for F^–^ in DMSO. Moreover, the anti-interference ability of the probe was evaluated by measuring the absorption spectra of fluorine ions in the presence of other competing ions. The selectivity of **4-PyOH** was studied by adding anions (Cl^−^, Br^−^, I^−^, HSO_4_^2−^, HSO_3_^2−^, S_2_O_3_^2−^, H_2_PO_4_^−^, AcO^−^, SO_4_^2−^, SO_3_^2−^, CrO_4_^2−^, NO_3_^−^, AC^−^, S^2−^, 200 *μ*M, respectively) in dry DMSO (Fig. S2). There was no clear absorption difference when competing ions were added to the probe solution. However, upon the addition of 30 *μ*M F^−^, absorption spectra presented an obvious expansion in 400–500 nm. The results indicated that **4-PyOH** was highly selective to F^−^ with complicated multi-ions' situation. With the change in the absorption spectra, the color of the **4-PyOH** solution changes from colorless to yellow in the presence of F^−^, showing that this “naked-eye” probe can be used for specificity determination for F^−^ as well.

In order to study the effect of acidity and alkalinity on the test, DMSO containing **4-PyOH** and PBS buffer solution with different pH values were mixed (*V*_DMSO_/*V*_PBS_ = 7 : 3) to obtain a **4-PyOH** stock solution (10 *μ*M). Based on the fluorescence spectra measurements recorded for the probe in different pH solutions (Figure S3), it was observed that as the pH gradually decreased, the fluorescence intensity of the probe at 525 nm diminished. In comparison, the fluorescence intensity near 385 nm increased and the fluorescence color transitioned from yellow to blue. Conversely, when the pH value reached 13.0, the fluorescence peak at 385 nm completely disappeared, and the solution's color deepened. A blue shift in the fluorescence spectrum was observed with decreasing pH, while an opposite red shift occurred with increasing pH. These spectral shifts were likely attributed to proton movement on the hydroxyl groups within the probe. However, within the pH range of 2.0–12.0, the probe exhibited relative stability and adaptability to complex acid-base environments. These findings suggested that the probe may find applications in diverse detection scenarios. To further investigate the FL stability of the probe, as well as with the F^−^ in THF, the FL intensity at 450 nm was continuously (0–10 min) measured every 30 s. The corresponding FL spectra and the variation tendency are summarized in Figure S4. The results showed that the intensity increased significantly in the initial 30 s after the addition of F^−^ and stabilized after about 2 min, implying a fast response speed of probe **4-PyOH** to F^−^.

The photostability is also crucial and is an important factor to measure the fluorescence properties of the compound. The fluorescence intensity of probe **4-PyOH** was tested with a three-purpose ultraviolet analyzer for different durations of irradiation. The test results are shown in Figure S5. With the illumination time ranging from 0 to 180 min, the fluorescence intensity of the compound basically did not change, indicating that the probe **4-PyOH** had good photostability.

To enlarge the applicable situation, the influence of temperature on the probe detection of F^−^ was investigated by proceeding with the detection in three temperature solutions (0°C, 20°C, and 40°C) and the results showed that temperature would not influence the detection effect (Figure S6). According to the thermogravimetric analysis (TGA) test, compound **4-PyOH** displayed a thermal decomposition (*T*_*d*_) temperature at 300°C (Figure S7), which implied a good probe thermal stability and longtime storage possibility.

### 3.2. Detection Mechanism

The recognition mechanism was postulated as follows: upon F⁻ detection, the probe initially establishes hydrogen bonds with fluoride ions. Subsequently, a deprotonation reaction induced by fluoride ions occurs, leading to the removal of protons from the phenolic hydroxyl group and the formation of phenolic oxygen anions. Similarly, due to the comparatively lower electron density at the 4-position of pyrene, the protons attached to the phenolic hydroxyl group at the 4-position are more prone to dissociation, rendering them more acidic. To validate the proposed reaction mechanism, ^1^H-NMR titration experiments and FTIR were conducted.

**Figure 2:**
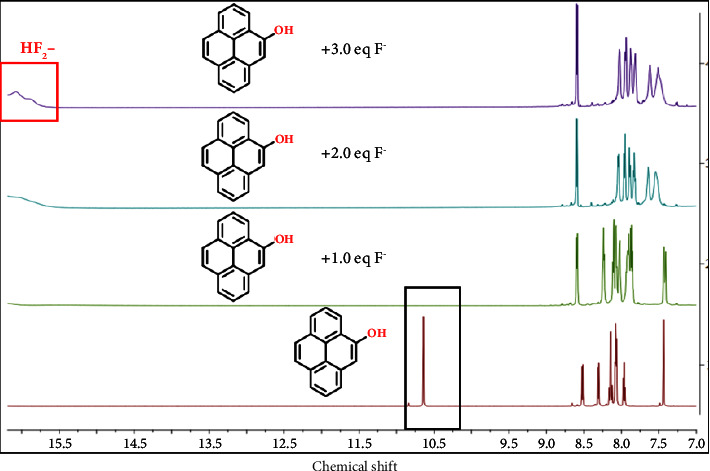
Nuclear magnetic resonance spectra were obtained by adding different equivalents of F^−^ to the *d*_6_-DMSO solution in which the probe **4-PyOH** was dissolved.

The F^−^ induced deprotonation mechanism between fluorine and the **4-PyOH** was further researched by titration of the ^1^H-NMR spectra of **4-PyOH** in *d*_6_-DMSO with different amounts of F^−^ (1.0 eq, 2.0 eq, and 3.0 eq). As shown in Figure 2, the ^1^H-NMR signal of the phenolic proton of **4-PyOH** (10.6 ppm) vanished following the addition of F^−^ (1.0 eq), but the typical peaks for other hydrogen remained unchanged. Further increase of F^−^, the mostly pyrenyl signals shifted upfield (dominated by a through-bond effect) [35] except that the one near the hydrogen bond (dominated by a through-space impact) [36] and a new signal located at 16.5 ppm corresponding to HF_2_^−^ could be observed. In the first step, a hydrogen-bonded species is formed between the F- and OH proton in the presence of lower equivalents of fluoride ions, followed by deprotonation at higher equivalents of fluoride ions. Hence, the results of the titration experiment indicated that **4-PyOH** adopted a hydrogen bond to interact with F^−^ at the phenolic OH group, and then F^−^ induced deprotonation to generate its phenolate anion. The sharp peak at 3001 cm^−1^ corresponds to OH stretching frequency in **4-PyOH** (Figure S8). Similarly, the OH stretching frequencies are broadened and enlarged due to the binding of fluoride ions to the hydroxyl hydrogen, which is reported that the hydrogen bonding interactions enhance the IR intensity for bands related to vibrational modes of functional groups directly involved in the hydrogen bonding [37].

### 3.3. Applications

To facilitate practical application, a disposable and portable **4-PyOH**-based test paper was developed. The test paper exhibited distinct color changes and alterations in the UV-vis spectra of **4-PyOH** upon exposure to increasing concentrations of F^−^ ions in organic solvents. This enabled the visual and quantitative detection of F^−^ ions using the test paper. The selective interaction between F^−^ ions and the hydroxyl group of **4-PyOH** caused the observed changes. The test paper demonstrated high sensitivity, a low detection limit, and minimal interference from other ions commonly found in organic solvents. The simplicity and convenience of the test paper offer a promising approach for on-site F^−^ ion detection in various applications such as environmental monitoring and industrial safety.

**Figure 3:**
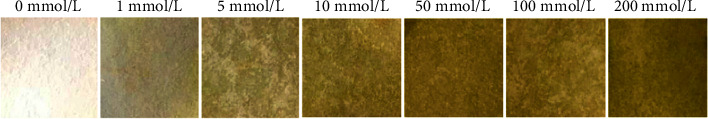
Test paper photographs for the monitoring of F^−^ in THF (different concentrations).

The **4-PyOH**-based test paper for monitoring F^−^ ions in THF via distinct color variation under daylight conditions is illustrated in Figure 3. The test paper was immersed in THF solutions with varying concentrations of F^−^ ions (ranging from 0 mmol/L to 200 mmol/L). Upon immersion, the test paper exhibited a gradual transition in color from colorless to yellow. The observed color change was directly correlated with the concentration of F^−^ ions in THF. Higher F^−^ ion concentrations resulted in more pronounced color changes. This distinctive and discernible color variation can be utilized as a visual pattern for quantitatively analyzing unknown F^−^ concentrations in THF. The **4-PyOH**-based test paper demonstrated its potential as a practical and efficient tool for on-site monitoring of F^−^ ions in THF. In order to better digital representation of the test paper color intensity change, each paper trip is photographed using Photoshop software to see its color value (R.G.B and Lab) and the data are summarized in Table S2. This approach offers a promising strategy for rapid and qualitative F^−^ ion detection in THF, with potential applications in various fields such as chemical synthesis, pharmaceutical manufacturing, and industrial processes.

**Figure 4:**
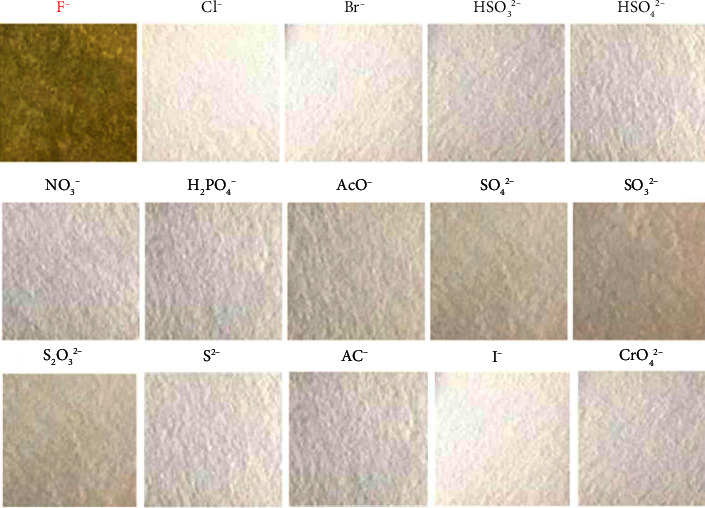
Images of test papers after being soaked in THF solution containing various anions in daylight.

The self-made cut test paper was dipped into a mixed solution containing different ions (Cl^−^, Br^−^, I^−^, HSO_4_^2−^, HSO_3_^2−^, S_2_O_3_^2−^, H_2_PO_4_^−^, AcO^−^, SO_4_^2−^, SO_3_^2−^, S_2_O_3_^2−^, CrO_4_^2−^, NO_3_^−^, AC^−^, S^2−^, ion concentration is 100 mmol/L) to observe the selectivity and sensitivity of the test paper towards F^−^. As shown in Figure 4, F^−^ caused evident color changes on the test paper, with a reaction time as short as approximately three seconds. No color changes were observed in other control experiments with different ions on the test paper. These experimental results demonstrated that the test paper can instantly and selectively detect F^−^ ions in THF (tetrahydrofuran) and holds potential for practical applications.

## 4. Conclusion

In summary, the fluorescent probe **4-PyOH** was synthesized by functionalizing the 4 positions of the pyrene core, enabling it to recognize trace amounts of F^−^ ions selectively. The probe **4-PyOH** demonstrated excellent selectivity and sensitivity for F^−^ ions, with a detection limit as low as 3.06 × 10^−7^ M. The presence of F^−^ ions can be easily identified with the naked eye using **4-PyOH**. Furthermore, the probe **4-PyOH** exhibited good stability and remained unaffected in solutions with a pH range of 2.0–12.0. Additionally, the probe **4-PyOH** was successfully incorporated into the test paper for detecting F- ions in organic solvents, yielding highly satisfactory results. The development of more novel fluorescent probes based on **4-PyOH** is anticipated, which could serve as a reference for future integration of multifunctional detection within a single probe molecule. In future work, based on the fluorescence characteristics of pyrene, more water-soluble probes were designed for the detection of various samples, including biological imaging. We believe that the molecular-level understanding of the sensing action and photophysical properties will facilitate the rational design of chemosensors.

## Data Availability

The data used to support the findings of this study are included within the supplementary information file.

## Supplementary Materials

Supplementary Materials Table S1: properties of the representative probes developed and the **4-PyOH** reported in this work. Figure S1: plot of FL intensity ratio of probe as a function of the concentration of F^−^ (a) for THF; (b) for DMSO (three parallel tests were conducted to get the error bar for detection effect description.). Figure S2: selective UV absorption spectra of **4-PyOH** (10 *μ*M) for various analytes. (F^−^ was 30 *μ*M, while Cl^−^, Br^−^, I^−^, HSO_4_^2−^, HSO_3_^2−^, S_2_O_3_^2−^, H_2_PO_4_^−^, AcO^−^, SO_4_^2−^, SO_3_^2−^, CrO_4_^2−^, NO_3_^−^, AC^−^, and S^2−^were 200 *μ*M, respectively). Figure S3: top: fluorescent photograph of **4-PyOH** (10 *μ*M) in DMSO: PBS = 7 : 3 (v/v) solution with increasing pH (pH = 0, 1, 2, 3, 4, 5, 6, 7, 8, 9, 10, 11, 12, 13). Bottom: the change of the fluorescence spectra of **4-PyOH** (10 *μ*M) in the solution of DMSO: PBS = 7 : 3 (v/v) at different pH values. Figure S4: the response change of FL intensity at 450 nm with time in the THF. Figure S5: PL spectra of compound **4-PyOH** in THF (a) and DMSO (b) with different UV irradiation durations. Figure S6: the change of the fluorescence spectra of **4-PyOH** (10 *μ*M) without (a) and with F^−^ (b) in solution of DMSO at different temperatures (0°C, 20°C, and 40°C). Figure S7: the thermogravimetric analysis curves of **4-PyOH**. Figure S8: FTIR spectra for **4-PyOH** and **4-PyOH** with F^−^. Table S2: color values for paper strips under F^−^content in THF.
