# Supplementary material for: Colorimetric and Fluorometric Determination of Fluoride in Tetrahydrofuran and Dimethyl Sulfoxide Using a 4-Hydroxypyrene Probe
Source: J Anal Methods Chem. 2024 Jul 26;2024:5566082. doi: 10.1155/2024/5566082 (PMC11300077; doi:10.1155/2024/5566082)
Supplement: Supplementary Materials — Table S1: properties of the representative probes developed and the 4-PyOH reported in this work. Figure S1: plot of FL intensity ratio of probe as a function of the concentration of F− (a) for THF; (b) for DMSO (three parallel tests were conducted to get the error bar for detection effect description.). Figure S2: selective UV absorption spectra of 4-PyOH (10 μM) for various analytes. (F− was 30 μM, while Cl−, Br−, I−, HSO42−, HSO32−, S2O32−, H2PO4−, AcO−, SO42−, SO32−, CrO42−, NO3−, AC−, and S2−were 200 μM, respectively). Figure S3: top: fluorescent photograph of 4-PyOH (10 μM) in DMSO: PBS = 7 : 3 (v/v) solution with increasing pH (pH = 0, 1, 2, 3, 4, 5, 6, 7, 8, 9, 10, 11, 12, 13). Bottom: the change of the fluorescence spectra of 4-PyOH (10 μM) in the solution of DMSO: PBS = 7 : 3 (v/v) at different pH values. Figure S4: the response change of FL intensity at 450 nm with time in the THF. Figure S5: PL spectra of compound 4-PyOH in THF (a) and DMSO (b) with different UV irradiation durations. Figure S6: the change of the fluorescence spectra of 4-PyOH (10 μM) without (a) and with F− (b) in solution of DMSO at different temperatures (0°C, 20°C, and 40°C). Figure S7: the thermogravimetric analysis curves of 4-PyOH. Figure S8: FTIR spectra for 4-PyOH and 4-PyOH with F−. Table S2: color values for paper strips under F−content in THF. [file 5566082.f1.docx]

**Supplementary material**

**Colorimetric and fluorometric determination of fluoride in tetrahydrofuran and dimethyl sulfoxide using a 4-hydroxypyrene probe**

Yue Sun^1,2^, Yunchen Long ^3^, Wenhao Sun ^2^, Yibo Zhang^3^, Qianhui Tang^4^, Chan Li ^1^, Sihua Li^1^，Jing Nie^1*,2^

^1^ Institute of Building Intelligence, Jiangsu Vocational Institute of Architectural Technology, Xuzhou, Jiangsu, 221116, PR China

^2^ School of Chemical Engineering and Technology, China University of Mining and Technology, Xuzhou, Jiangsu, 221116, PR China

^3^ Department of Materials Science and Engineering, City University of Hong Kong, Hong Kong, China

^4^Environmental Engineering, Dalian Ocean University, Dalian, Liaoning, 116023, PR China

**Table of contents**

**Table S1.………………………………………………………...............................S3-4**

**Fig. S1-S2………………………………………………………...............................S5**

**Figs. S3-S4…………………………………………….………………………........S6**

**Figs. S5-S6………………………………………………………………………......S7**

**Table S2, Figs. S7-S8………………………………………………………………..S8**

**References…………………………………………….…………………………......S9-14**

**Table S1.** Properties of the representative probes developed and the **4-PyOH** reported in this work.

| Ref. | Core structure | Probe Type | Maximumemission wavelength with F− | Application |
| --- | --- | --- | --- | --- |
| This work | **4-hydroxypyrene probe**  **4-PyOH** | Colorimetric method and  Turn on | 500 nm in THF  550 nm in DMSO | As test strips, colorimetric and fluorometric determination of fluoride in tetrahydrofuran and dimethyl sulfoxide |
| 1 | Phenothiazine derivatives  **LDT** | Turn on | 450 nm in THF | Sensitive detecting  Fluoride ions |
| 2 | Furan/thiophene-based fluorescent hydrazones | Turn off | 520 nm in THF | As test strips, detecting fluoride ions and cyanide ions |
| 3 | Adenine-linked naphthalimide  **Conmpond 1** | Colorimetric method | -- | Colorimetrically detects F− ions over a series of other anions in CH_3_CN containing 1%DMSO |
| 4 | coumarin-based fluorescent probes **1and 2** | Turn on | Probe 1 ：  472 nm in CH_3_CN  Probe 2:  450 nm in DMSO/HEPES buffe(v/v=1:9) | The detection of  fluoride ions in organic solvent and in cells |
| 5 | a carbazole‑based fuorescent probe **SCC** | Turn on | 510 nm in IPA | Detect fuoride ions in aqueous systems |
| 6 | Chalcone **1a** | Colorimetric method | -- | Detection of fluoride and cyanide ions |
| 7 | *meso*-substituted BODIPYs derivatives  **B1** | ON-OFF | 515 nm in chloroform | Detection of fluoride ions in polar aprotic organic solvents |
| 8 | Schiff base fluorescent probe **L** | Colorimetric and turn-off | 514 nm in dmso | Detection of fluoride ions in aqueous medium |
| 9 | triphenylsilyl-containing gelator | gelator-nongelator | -- | Detection of fluoride ions |
| 10 | small-molecule gelator **compound** **2** | gelator-nongelator | _ | visual detection of fluoride ion |
| 11 | naphthalimide-based probe **L** | Colorimetric and turn-off | 550 nm in DMSO-water (v/v=7:1) | Detection of fluoride ions in DMSO-water (v/v=7:1) solution. |
| 12 | 4-aminophenylboronic acid dimer (SPCE*/APBAD) | Electrochemical method | _ | Detection of fluoride ions |
| 13 | diaminomaleonitrile-derived Schiff base compound **1-4** | Colorimetric  Nake-eye | -- | Highly Selective  detection of fluoride ions in THF and water |


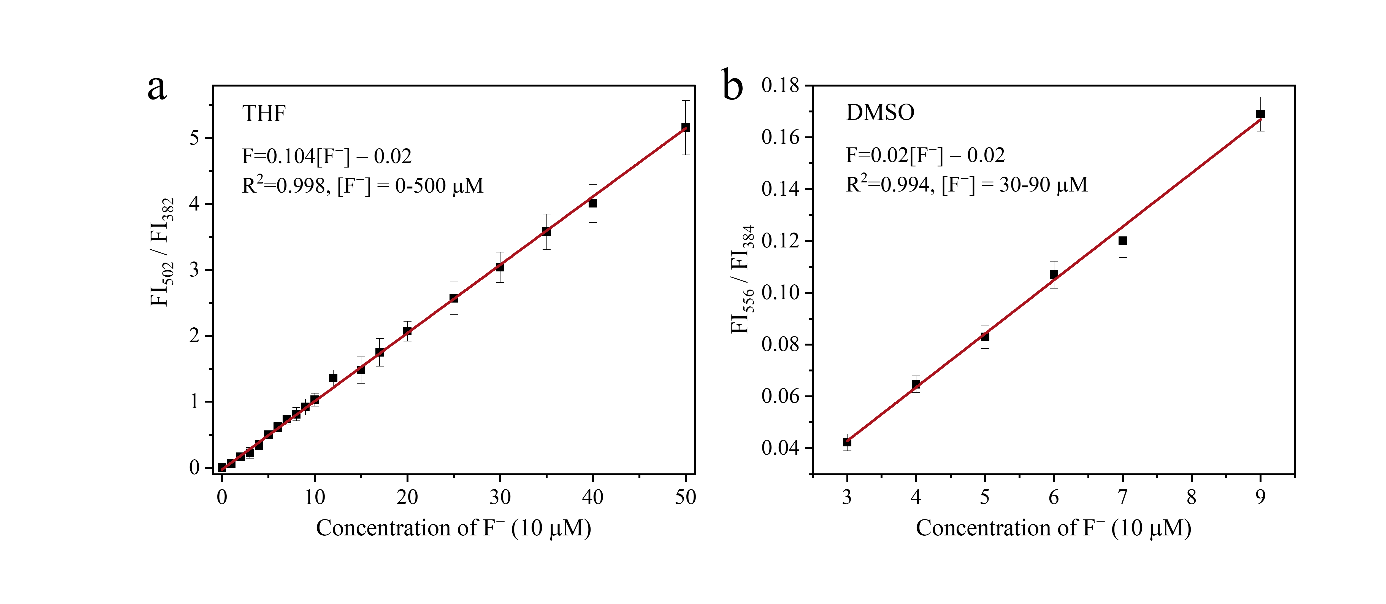


Fig S1 Plot of FL intensity ratio of probe as a function of the concentration of F^−^ a). for THF. b) for DMSO. (Three parallel test were conducted to get the error bar for detection effect description.)


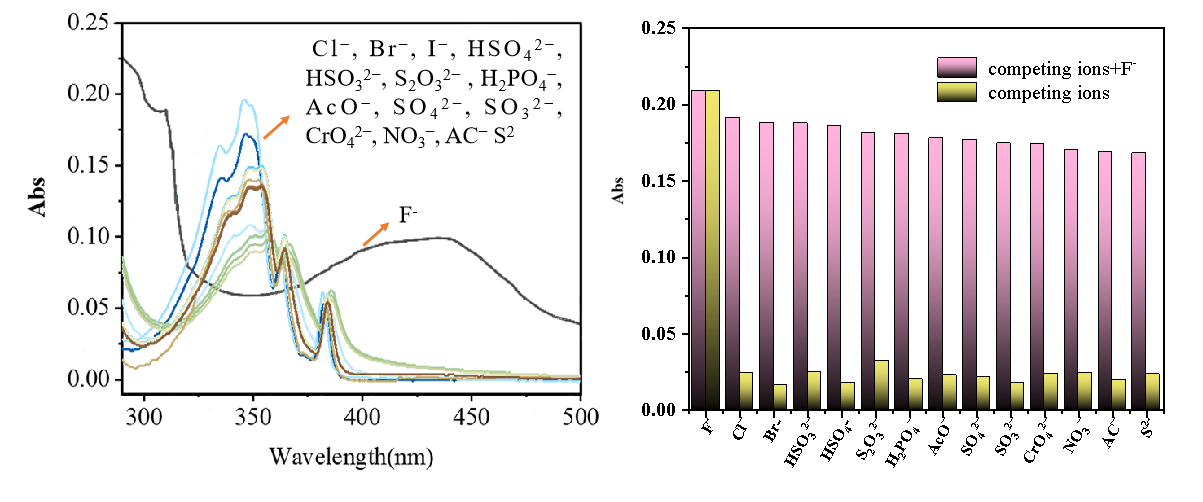


Fig. S2 Selective UV absorption spectra of **4-PyOH** (10 μM) for various analytes. (F^−^ was 30 μM, while Cl^−^, Br^−^, I^−^, HSO_4_^2−^, HSO_3_^2−^, S_2_O_3_^2−^ , H_2_PO_4_^−^, AcO^−^, SO_4_^2−^, SO_3_^2−^, CrO_4_^2−^, NO_3_^−^, AC^−^ S^2−^were 200 μM, respectively).


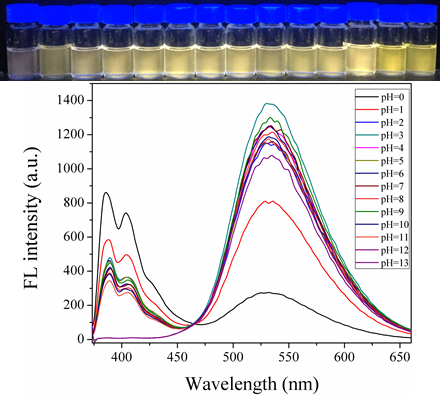


Fig. S3: Top: Fluorescent photograph of **4-PyOH** (10 μM) in DMSO: PBS = 7: 3 (v / v) solution with increasing pH (pH = 0, 1, 2, 3, 4, 5, 6, 7, 8, 9, 10, 11, 12, 13). Bottom: The change of the fluorescence spectra of **4-PyOH** (10 μM) in the solution of DMSO: PBS = 7: 3 (v / v) at different pH values.


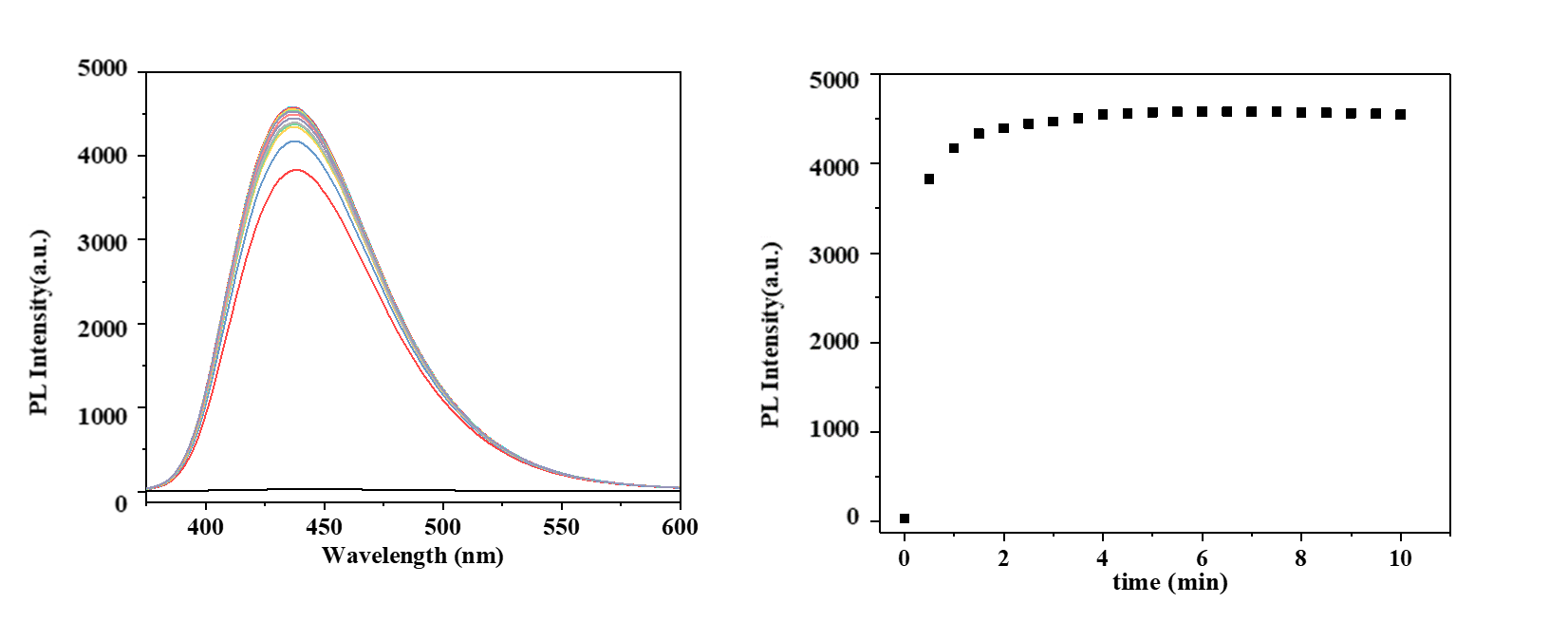


Fig. S4 The response change of FL intensity at 450 nm with time in the THF


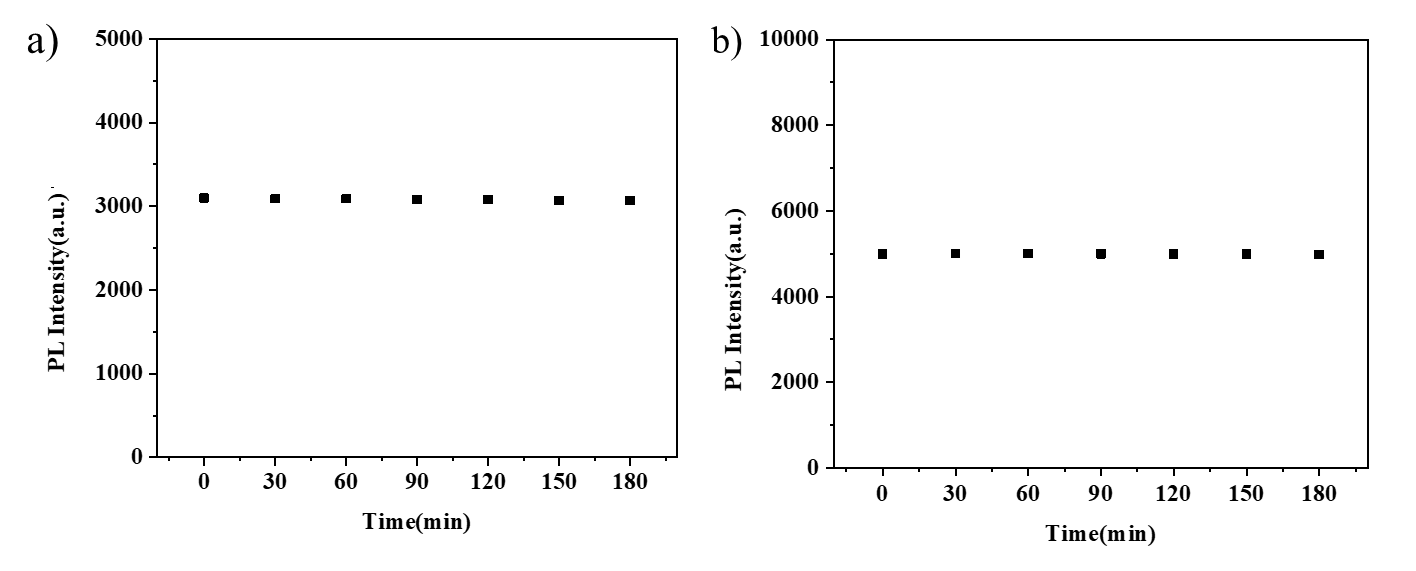


Fig. S5 PL spectra of compound **4-PyOH** in THF (a) and DMSO (b) with different UV irradiation durations


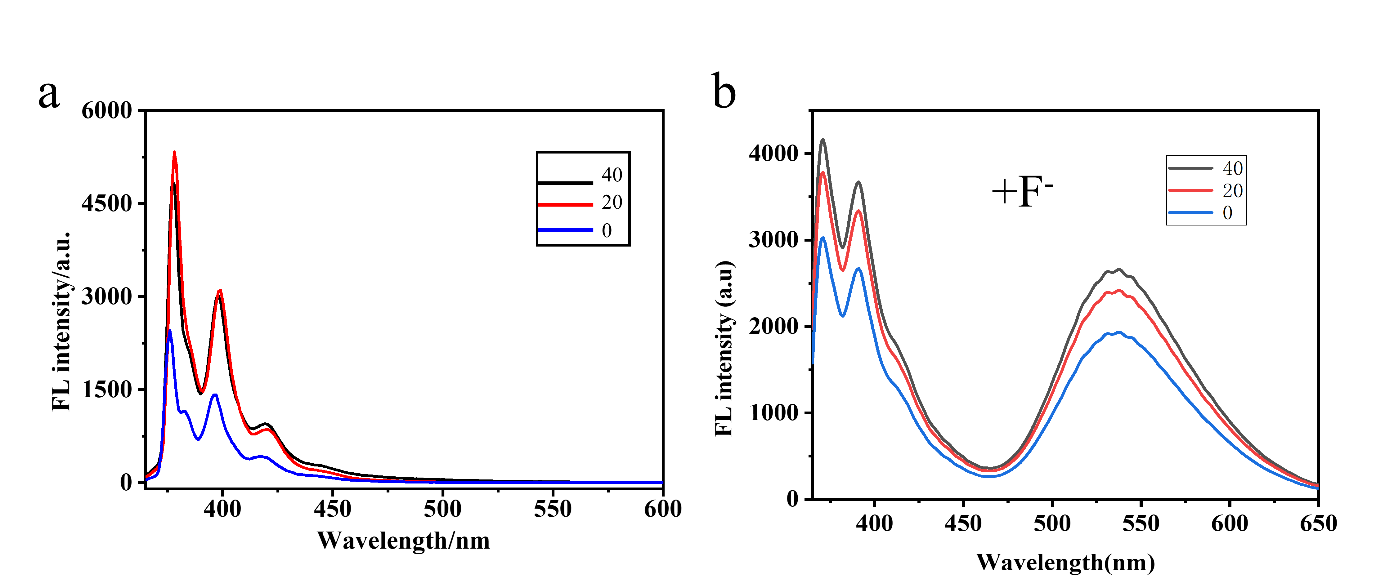


Fig. S6 The change of the fluorescence spectra of **4-PyOH** (10 μM) without(a) and with F^−^ (b) in solution of DMSO at different temperatures (0 ℃, 20℃, 40℃).

Fig. S7 The Thermogravimetric analysis curves of **4-PyOH**

Fig. S8 . FTIR spectra for **4-PyOH** and **4-PyOH** with F^−^

Table S2 Color values for paper stips under F^−^content in THF

| F^−^content in THF | 0 mmol/L | 1 mmol/L | 5 mmol/L | 10 mmol/L | 50 mmol/L | 100 mmol/L | 200 mmol/L |
| --- | --- | --- | --- | --- | --- | --- | --- |
| R.G.B | 251 247 238 | 187 173 136 | 176 157 76 | 172 148 48 | 167 144 45 | 167 144 45 | 157 137 13 |
| Lab | 96 1 4 | 73 -1 19 | 72 -2 32 | 65 0 42 | 60 1 54 | 58 0 50 | 47 0 52 |

**References:**

1. Feng, T.; Yang, J.; Tu, S.; Yang, T.; Wu, T.; Zhu, W.; Le, Y.; Liu, L. Design, Synthesis, and Cellular Imaging Application of a Fluorescent Probe Based on Fluoride Ion-Induced Cyclization of Phenothiazine Derivatives. Journal of Fluorescence 2023. DOI: 10.1007/s10895-023-03526-3.
2. Saini, N.; Wannasiri, C.; Chanmungkalakul, S.; Prigyai, N.; Ervithayasuporn, V.; Kiatkamjornwong, S. Furan/thiophene-based fluorescent hydrazones as fluoride and cyanide sensors. Journal of Photochemistry and Photobiology A: Chemistry 2019, 385. DOI: 10.1016/j.jphotochem.2019.112038
3. Pati, C.; Raza, R.; Ghosh, K. Adenine-linked naphthalimide: A case of selective colorimetric as well as fluorometric sensing of F− and anion-activated moisture detection in organic solvents and CO2-sensing. Spectrochimica Acta Part A: Molecular and Biomolecular Spectroscopy 2020, 229. DOI: 10.1016/j.saa.2019.117910.
4. Xu, X.; Chen, Y.; Wei, L.; Mao, W.; Lin, F.; Zhou, X. Fluorescent turn-on probes for the detection of fluoride ions in organic solvent and in cells. Analytical Methods 2016, 8 (2), 245-248. DOI: 10.1039/c5ay02267c.
5. Li, D.; Tu, S.; Le, Y.; Zhou, Y.; Yang, L.; Ding, Y.; Huang, L.; Liu, L. Development of a carbazole-based fluorescent probe for quantitative detection of fluoride ions in aqueous systems. Chemical Papers 2023, 77 (3), 1741-1749. DOI: 10.1007/s11696-022-02557-7.6
6. Maurya C K , Gupta P K .Discriminative Chromogenic Detection of Fluoride and Cyanide Ions[J].Chemistry Select, 2024(3):9. DOI**：**10.1002/slct.202304016
7. Mathew, D.; Arunkumar, C.; Sujatha, S.; Parameswaran, P. Hydrogen-bonding receptor substituted BODIPYs as selective ON-OFF fluorimetric sensors for fluoride ions in polar aprotic organic solvents – A molecular-level understanding based on experimental and theoretical studies. Journal of Photochemistry and Photobiology A: Chemistry 2023, 442. DOI: 10.1016/j.jphotochem.2023.114780.
8. Devi, B.; Guha, A. K.; Devi, A. Fluoride ion detection in aqueous medium: Colorimetric and turn-off fluorescent Schiff base chemosensor. Spectrochimica Acta Part A: Molecular and Biomolecular Spectroscopy 2024, 305. DOI: 10.1016/j.saa.2023.123448.
9. Singh, W. P.; Singh, R. S. A new triphenylsilyl-containing gelator for visual sensing of fluoride ion. Materials Chemistry and Physics 2020, 241. DOI: 10.1016/j.matchemphys.2019.122351.
10. Singh, W. P.; Singh, L. R.; Singh, R. S. Gelation–based visual detection of fluoride ion: Strategic use of silyl protection–deprotection chemistry. Journal of Molecular Liquids 2022, 360. DOI: 10.1016/j.molliq.2022.119536.
11. Xiao, L. , Ren, L. , Jing, X. , Li, Z. , & Guo, D. . (2019). A selective naphthalimide-based colorimetric and fluorescent chemosensor for "naked-eye" detection of fluoride ion. Inorganica Chimica Acta, 2019, 119207. d DOI:10.1016/j.ica.2019.119207
12. Thiruppathi, M.; Natarajan, T.; Zen, J.-M. Electrochemical detection of fluoride ions using 4-aminophenyl boronic acid dimer modified electrode. Journal of Electroanalytical Chemistry 2023, 944. DOI: 10.1016/j.jelechem.2023.117685.
13. Ullah, Z.; Subramanian, S.; Lim, H.; Dogan, N. A.; Lee, J. S.; Nguyen, T. S.; Yavuz, C. T. Highly Selective and Scalable Molecular Fluoride Sensor for Naked-Eye Detection. ACS Applied Materials & Interfaces 2024. DOI: 10.1021/acsami.4c01187.
